# Batch-fabrication of all-dielectric vapor cells enabling optically addressed Rydberg atom electrometry

**DOI:** 10.1038/s41378-026-01221-4

**Published:** 2026-06-18

**Authors:** Alexandra B. Artusio-Glimpse, Adil Meraki, Hunter Shillingburg, Guy Lavallee, Miao Liu, Chad Eichfeld, Nikunjkumar Prajapat, Matthew T. Simons, Glenn Holland, Christopher L. Holloway, Vladimir A. Aksyuk, Daniel Lopez

**Affiliations:** 1 Electromagnetic Fields Group, National Institute of Standards and Technology, Boulder, 80305 CO USA; 2 Physics Department, University of Colorado Boulder, Boulder, 80303 CO USA; 3 Electrical Engineering and Computer Science, Penn State University, University Park, 16802 PA USA; 4 Materials Research Institute, Penn State University, University Park, 16802 PA USA; 5 Microsystems and Nanotechnology Division, National Institute of Standards and Technology, Gaithersburg, 20899 MD USA

**Keywords:** Optical materials and structures, Optical sensors

## Abstract

Millimeter-scale atomic vapor cells can be accurately and economically batch-fabricated by anodically bonding silicon and glass wafers, enabling the manufacturing of miniature atomic clocks and quantum sensors. However, silicon’s high dielectric constant and conductive losses at millimeter wave frequencies limit its suitability for Rydberg-atom electrometry, which enables highly sensitive electric-field measurements by exploiting the extreme polarizability of Rydberg states in alkali atoms. To address this, we present an all-glass wafer-level microfabrication process that eliminates silicon, creating hermetically sealed vapor cells that are stable over long timelines with embedded cesium dispensers. Femtosecond laser machining precisely defines the cell geometry, and laser-activated alkali loading ensures reliable filling. We demonstrate long-term vacuum stability and robust Rydberg excitation through electromagnetically induced transparency measurements. We then use these cells to measure a 34 GHz millimeter wave field resonant with the $58{{\rm{D}}}_{5/2}\to 60{{\rm{P}}}_{3/2}$ transition using Autler-Townes splitting and observe the expected linear dependence with field strength. This work demonstrates that the all-glass bonding approach offers a highly durable low-loss cell alternative for miniaturized millimeter wave and microwave quantum sensing, with the potential to flexibly incorporate a range of other dielectric and semiconductor materials and integrate with photonic and electronic technologies.

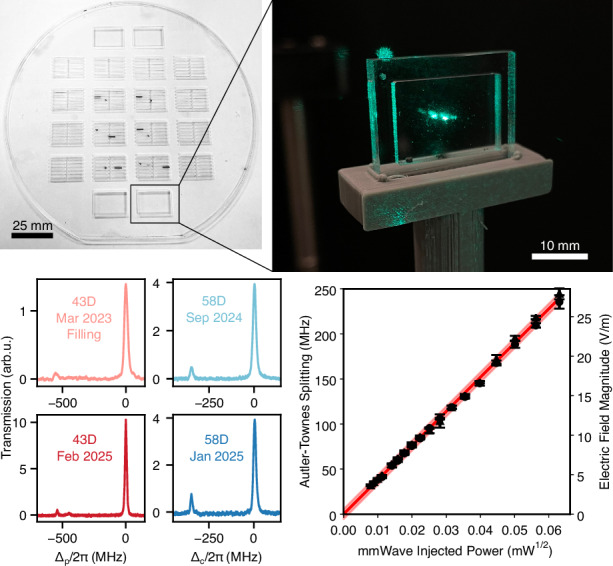

## Introduction

Miniaturized alkali-vapor cells have enabled a range of precision measurement technologies, including global positioning systems, telecommunications, chip-scale magnetometers, and, more recently, broadly tunable Rydberg-atom electric field sensors. Over the last two decades, as these applications have diversified, a variety of fabrication techniques have emerged, each optimized for a different operating regime. The most prominent methods include glass-blown vapor cells at the millimeter scale and anodically bonded glass-and-Si chip-scale vapor cells, both of which allow batch fabrication. Elsewhere, heterogeneous integration of diverse material platforms, such as III-V semiconductors and glass substrates, has shown great promise for high-speed electronics and advanced integrated photonics. This integration is often achieved through direct bonding, where cleaned, polished, and activated surfaces are brought into contact and annealed at modest temperatures to form strong covalent bonds^1,2^. As chip-scale vapor cells are integrated with photonics systems^3–8^, new compatibility challenges arise, driving the need for new cell materials, structure geometries, and innovative fabrication processes.

Rydberg atom electrometry is a technique that uses the extreme sensitivity of Rydberg atoms to electric fields for precise measurements^9^. Alkali atoms, excited to high principal quantum numbers, exhibit exaggerated electromagnetic coupling, making them ideal for optically detecting and measuring electric fields with high accuracy across an extended and tunable range of frequencies. Detection of over-the-air millimeter wave (30–300 GHz) signals using Rydberg atoms presents new challenges and opportunities for optimizing the incident field interactions with the vapor cell materials, structured at the millimeter scale comparable to the RF wavelength. Combined with the need for photonic integration of the optical fields for excitation and read-out of Rydberg atoms, we were motivated to develop a direct bonding wafer-level fabrication process for small vapor cells that offers broader material options. The stable high vacuum hermetic seal critical to a wide range of cell applications is stringently tested by ground state and Rydberg state spectroscopy measurements.

Glass-blown alkali cells are produced in sizes ranging from several centimeter cylinder and spherical cells for laboratory spectroscopy down to 3–5 mm spheres for satellite clocks and compact magnetometers and gyroscopes. Below roughly 5 mm, the process leads to measurable birefringence and surface curvature, imperfections that must be mitigated by post-blow annealing or polarimetric compensation^10^. Hollow-core photonic-crystal fibers and related silica waveguides offer a complementary route to miniaturization. Alkali vapor can be loaded into 5 ${\rm{\mu }}{\rm{m}}$ to 150 ${\rm{\mu }}{\rm{m}}$ cores, giving centimeter-long interaction lengths and large optical depths suitable for a range of sensing applications. However, a practical consideration is that vapor fills the fiber axially, so local density variations can introduce static field gradients along the fiber length, an effect that must be managed in high-precision Rydberg measurements^11,12^.

Millimeter-scale vapor cells used in atomic clocks^13,14^, optically pumped magnetometers^15^, and absolute gravity sensors^16^, on the other hand, are accurately batch-fabricated from silicon and borosilicate glass wafers using optical lithography and anodic bonding^17,18^. However, anodic bonding typically requires doped silicon, typical resistivity in the 1 $\Omega \cdot {\rm{cm}}$–30 $\Omega \cdot {\rm{cm}}$ range^19^, a material with high dielectric permittivity and conductive loss for radio frequency (RF) fields^20^. The resulting strong absorption, scattering, and local perturbation of the incident RF fields are undesirable for Rydberg atom-based RF measurements and may be detrimental in other atomic quantum sensing scenarios.

Rydberg-atom-based measurements of microwave fields have been shown in silicon-based anodically bonded vapor cells^21,22^; however, improved field sensitivity is expected with more RF-friendly cell materials. Thinning the silicon walls is one option to minimize the undesired field-structure interaction^23^. Alternatively, sputtered thin-film silicon^24^ can be used in anodic bonding with borosilicate or aluminosilicate glasses to minimize the volume of silicon in the vapor cell. Subwavelength silicon patterning has been proposed for engineering the dielectric constant to minimize scattering of the RF field^25,26^. Such dielectric structure engineering can be further optimized if a broader choice of cell materials became available. High-resistivity intrinsic silicon can be bonded to glass using plasma surface treatment^27^. The most promising technique for batch-manufacturing anodically bound vapor cells from high-resistivity silicon involves the deposition of Al_2_O_3_^28^, which acts as an adhesion layer^29^ and reduces the consumption of alkali^30^. But, regardless of the bonding technique used, the high dielectric constant of intrinsic silicon at $\approx 11.7$^31^ compared to that of fused silica at $\approx 4$ and Borofloat 33^32^ at $\approx 4.5$^33^, presents a much stronger spatially dependent perturbation of the electric field measured by the atoms, which is undesirable for many millimeter wave applications. Therefore, all-glass vapor cells for these applications are preferred.

A small number of miniature glass vapor cells have been reported using bonding techniques such as glass fusion^34^, optical contact bonding^35,36^, edge welding^37^, epoxy glue^38,39^, hot wire cutting^40^, and even anodic bonding via a 200 nm thick sputtered layer of silicon nitride^41^. Although these demonstrations of small, even micrometer-sized vapor cells prove that such miniature vapor cells can host Rydberg atoms for a range of applications, the fabrication schemes are not easily scalable to batch-fabrication with the exception of the silicon nitride anodic bonding method.

Here, we demonstrate the feasibility of using direct bonding for wafer-scale manufacturing of high-performance alkali-vapor cells, expanding the toolkit for integrating atomic vapor with photonics, lasers, electronics, and mechanical devices. By successfully fabricating and filling glass vapor cells with long observed lifetimes, our work broadens the range of materials available for mass manufacturing and wafer-level integration of atomic vapor, paving the way for a new generation of heterogeneously integrated systems. Borofloat 33 substrates are used because of the familiarity of this material in many vapor cell systems and because of their low dielectric constant and low loss in the RF domain. With this demonstrated microfabrication of high-quality direct-bonded cells using a commercial 150 mm wafer bonding tool, we overcome current fabrication limitations and pave the way for many more materials to be used in miniature atomic vapor systems.

We detail electromagnetically induced transparency (EIT) measurements using cesium (Cs) Rydberg atoms, carried out repeatedly over a period of 23 months, indicating good performance over a long period. Comparisons of cells from different wafer batches suggest the reliability of this manufacturing process. We carry out Rydberg atom electrometry of a 34 GHz electric field using the 58D_5/2_ → 60 P_3/2_ resonant transition as a test case to demonstrate the applicability of this cell manufacturing technique for the detection of millimeter wave fields. Using wafer-level femtosecond laser machining, we accurately create intricate cavity geometries in all-dielectric cells that can be tailored to minimize or custom-engineer near- and far-field scattering of the RF field, while avoiding deleterious absorption, resulting in a tailorable and more robustly predictable relationship between the RF fields outside and inside the vapor cell over a wide range of frequencies. The presented cells with approximately 1.9 × 1.3 × 0.2 cm^3^ cavities have been engineered specifically for the future goal of performing sub-wavelength measurements and mapping of a millimeter wave field using the Rydberg atoms at multiple spatial locations simultaneously.

## Results

### Fabrication and filling process

**Fig. 1:**
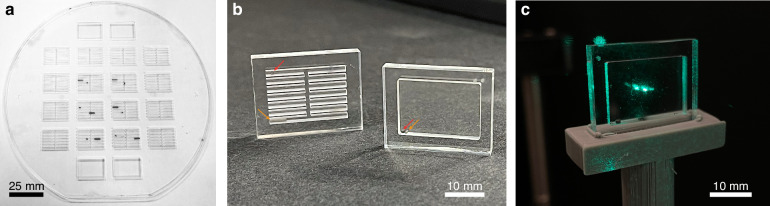
Wafer-level manufactured vapor cells. Photographs of (**a**) a bonded 150 mm diameter wafer having 20 vapor cells with different geometries, six of which in this case contain a cesium dispenser and a getter, **b** two filled vapor cells - a supported version and an open version (red arrows point to Cs dispensers and orange arrows point to getters), and **c** an open cell shown with the Rydberg lasers propagating through it

Our wafer-level vapor cells are fabricated by bonding a triple-stack of borosilicate wafers to form a full 150 mm wafer of twenty vapor cells, as shown in Fig. 1a–c. Bonded stacks are diced into individual vapor cell die with nominal outer dimensions of $25\times 20\,\mathrm{mm^2}$. The thickness of the middle wafer, which defines the length of the optical path within the vapor cell, is 2 mm and the thickness of each of the window wafers is 1 mm. The internal cavity volume of each vapor cell is machined through the middle wafer prior to bonding using femtosecond laser pulses and potassium hydroxide (KOH) etching, resulting in a variety of internal cell geometries tailored to the application needs. The KOH etch is highly selective to the denatured glass areas defined by femtosecond laser exposure, preserving the surface quality of the bond interface without need for additional polishing. Throughout this paper, we discuss measurements with two internal cell geometries, supported and open, as pictured in Fig. 1b and discussed in more detail in Section “Vapor cell geometry details”. Notably, these cells come from different wafer batches, the supported cell bonded in January 2023 and the open cell bonded in September 2024, which highlights the reliability and repeatability of this manufacturing process.

Further miniaturization is feasible with this wafer-level process; in practice, the minimum lateral footprint can be pushed down toward the alkali source pill size. The main limiting factor is therefore not the bonding/etching, but reliable handling, placement, and sealing of very small source pieces under vacuum while maintaining clean, hermetic interfaces.

**Fig. 2:**
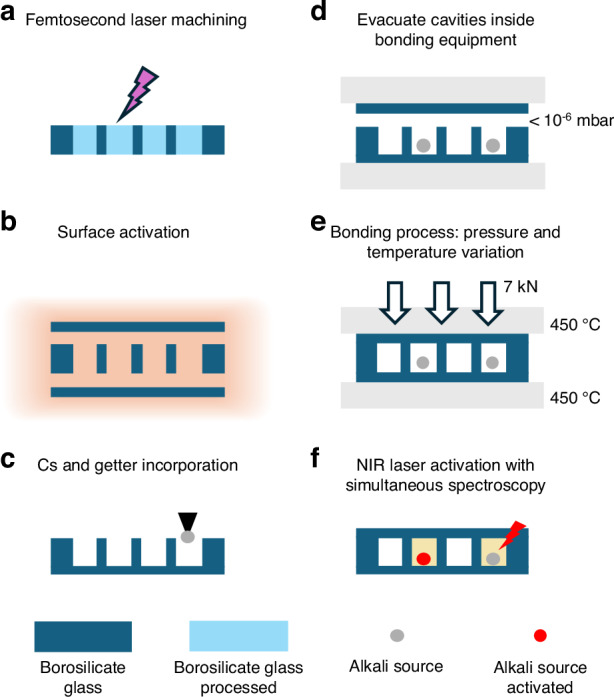
MEMS vapor cell fabrication process. **a** femtosecond (fs) laser machining of the 2 mm thick glass wafer, **b** cleaning and surface activation of glass wafers before bonding of the preform as depicted in (**e**), **c** placement of the alkali dispensers and non-evaporable getters, **d** introduction into bonding equipment and evacuation of the bonding chamber down to ${10}^{-6}$ mbar, **e** application of pressure at elevated temperature for 20 h, and **f** activation of the alkali source pills by near-infrared (NIR) laser heating. Wafer dicing to separate each vapor cell is performed either prior to or just after step (**f**)

The wafer fabrication sequence is depicted in Fig. 2. Following the machining of the glass frame (middle wafer), the machined wafer and both the top and bottom window wafers are placed in a 75° sulfuric acid/hydrogen peroxide bath for 1 h, which cleans and activates the surfaces of the wafers. After removal from the chemical bath, the wafers are rinsed with deionized water and dried using N_2_ in a spin-rinse dryer. After drying, the bottom window wafer and the machined middle wafer are brought into contact to form a partially sealed cell (a preform). Next, placement of a Cs dispenser (Cs-56Zr-11Al) and a non-evaporable getter (NEG) within each cell cavity is performed using a vacuum pen to minimize particle deposition on the bond surface. The NEG is added for additional background gas pumping, if needed. Immediately after placing the Cs dispensers and getters in the cells, the partially sealed cells and the top window wafer are loaded into the bonding equipment. The time between the clean step and wafer contact for bonding must be 30 min or less for a successful bond to occur.

The bonding process starts by evacuating the system to a base pressure of $< {10}^{-6}{\rm{mbar}}$ while at the same time keeping the bonding substrates separated to ensure that the completed vapor cells will be sufficiently evacuated after bond. Once the appropriate vacuum level is achieved, the wafers are then brought together and heated to a temperature between 450 and 500 °C with a force of $\approx 7\,\mathrm{kN}$ applied to the wafers. After holding these conditions for 20 h, the temperature is then set to 20 °C, and the applied pressure is turned off so that the wafers are allowed to cool and relax naturally.

**Fig. 3:**
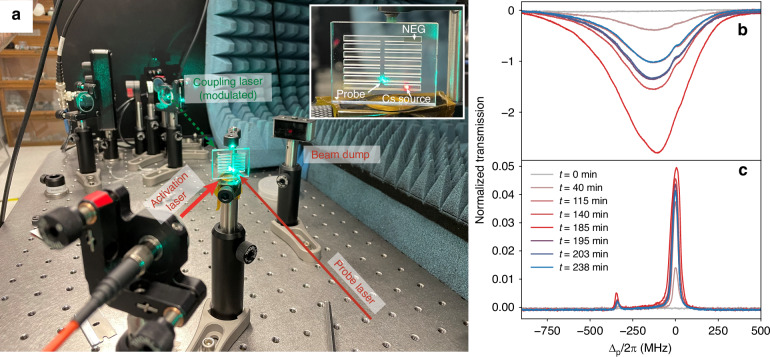
Vapor cell characterization. **a** Dispenser activation setup including inset picture of a wafer-processed glass vapor cell (red light on Cs dispenser is guide beam coaligned with the $\approx 1$ W, 980 nm activation laser). Plots on right show simultaneous **b** absorption and **c** 50D_5/2_ EIT measurements over time as the Cs dispenser was activated. Activation laser turned off at $t=201\min$ after which the vapor cooled and stabilized. Data has been smoothed for improved visibility

Upon completion of the bonding, each vapor cell is diced out of the wafer stack and filled by laser-heated activation of the Cs dispensers; the order between filling and dicing is interchangeable. Laser-heated activation of the getters was completed in some of the vapor cells, but it was not found to have a measurable impact on the measured spectra, suggesting evacuation in the bonding tool was sufficient (see Section “EIT spectra and RF electrometry” for further analysis of the background pressure in one of these vapor cells). During laser activation, a two-photon Rydberg EIT signal is monitored while scanning the probe laser over the D2 Doppler absorption profile. See Fig. 3 for example data taken from one of these test cells monitoring the 50D_3/2_ and 50D_5/2_ Rydberg states. We used Cs dispensers in this proof-of-concept study because they are common alkali sources in microfabricated vapor cells. However, the Zr-Al scaffolding left behind as a pill (also a getter) after Cs release may perturb incident RF fields. Future iterations will explore alternative filling processes, such as using pure alkali metals or methods that separate the alkali source from the final vapor cell.

**Fig. 4:**
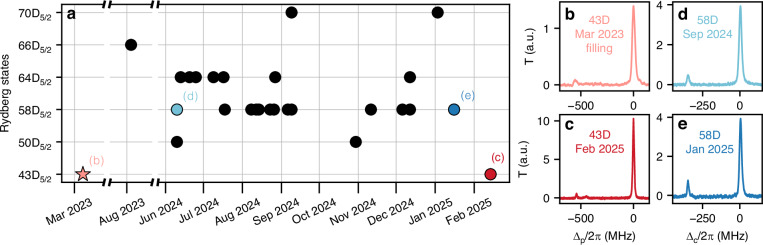
Timeline of Rydberg EIT measurements. **a** Dates of Cs filling by laser-heated activation (star) and all Rydberg EIT spectroscopy measurements collected with one supported vapor cell (circles) from a wafer lot bonded in Jan 2023. **b**–**e** Selection of recorded EIT spectra corresponding to labeled markers in (**a**)

The first batch of vapor cells using this direct bonding process and laser-heated dispenser activation was bonded on 25 January 2023, the activation study was completed on 6 March 2023, and regular measurements of Rydberg EIT spectra until the writing of this paper confirm that the cells have maintained original background pressure and filling levels for almost 2 years. Figure 4a shows a timeline of Rydberg EIT measurements collected with a single vapor cell from this first batch (a supported version) up to the time of writing this paper. The Rydberg state of each measurement is denoted on the vertical axis. Each point represents a day when Rydberg EIT signals were observed, each under different experimental conditions that prohibited a quantitative comparison between these datasets; however, the strong EIT signals portrayed in Fig. 4b–e support the conclusion that this cell is well sealed. The lifetime of this vapor cell is 707 days and counting.

### EIT spectra and RF electrometry

**Fig. 5:**
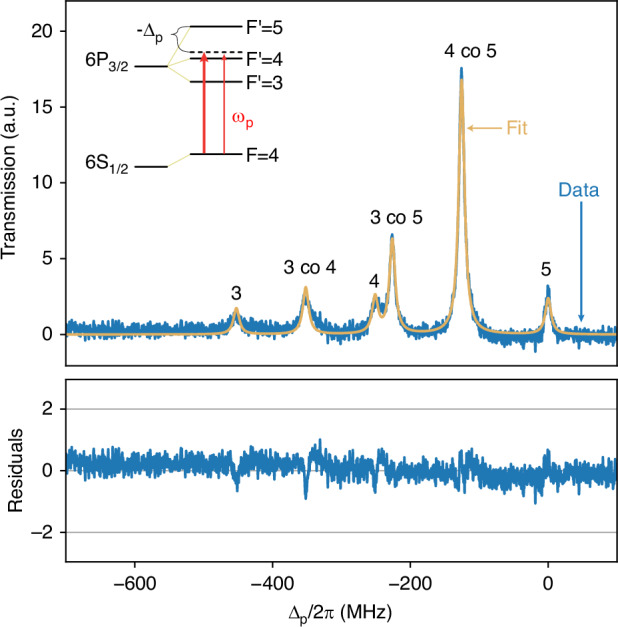
Saturated absorption spectrum of the Cs D2 line with the Doppler background subtracted. The fit (Eq. 1 in ref.^42^) predicts the product ${\varGamma }_{{vcc}}{\tau }_{R}=0.00\left(2\right)$, consistent with zero, confirming adequate evacuation of the vapor cell prior to filling. Reported uncertainty is 95% confidence in the fit coefficient

Before examining the EIT signals, we first characterize the atomic vapor cell using saturated absorption spectroscopy (SAS), as shown in Fig. 5a. This technique allows us to resolve the hyperfine structure of the D2 ground-state transition. The well-defined peaks corresponding to different hyperfine transitions validate the cell’s functionality and ensure the vapor cell was sufficiently pumped down to minimize background pressure prior to filling^42^. In this measurement, the probe beam power was $10{\rm{\mu }}{\rm{W}}$ with a beam width of $867\,{\rm{\mu }}{\rm{m}}$ and the pump and probe beams were aligned counter-propagating. A fit is applied to this spectrum (Eq. 1 in ref. ^42^), which involves 12 free fit parameters: six amplitudes (one for each Lamb dip peak), the homogeneous linewidth (common to all peaks), temperature, the unitless produce ${\varGamma }_{{vcc}}{\tau }_{R}$ defined by the rate of velocity changing collisions and the relaxation rate of the ground state, and three parameters used to account for any nonlinearity or frequency shifts in the frequency axis. The product ${\varGamma }_{{vcc}}{\tau }_{R}\propto p$, where $p$ is the pressure of some deleterious gas, is extracted from a fit to all six Lamb dips. We find from this fit that ${\varGamma }_{{vcc}}{\tau }_{R}=0.00\left(2\right)$, consistent with zero, which confirms adequate evacuation of the vapor cell prior to filling. The line width of $\varGamma =10.03\left(1\right){\rm{MHz}}$ includes contributions from the natural lifetime of the P state, power broadening, and a slight broadening effect caused by a known magnetic field $< 0.1{\rm{mT}}$ aligned with the polarization axis of the lasers.

**Fig. 6:**
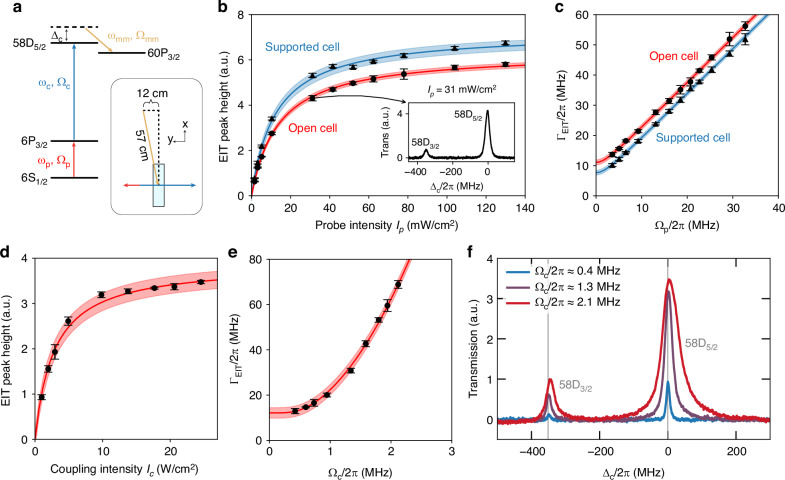
Electromagnetically Induced Transparency spectrum. **a** Energy diagram corresponding with this figure and Fig. 7. Each field frequency (${\omega }_{i}$), Rabi frequency (${\varOmega }_{i}$), and detuning (${\Delta }_{i}$) are reported in the text, where $i=P,C,{mm}$ refer to probe, coupling, and millimeter wave fields, respectively, and ${\Delta }_{P}$ = ${\Delta }_{{mm}}=0\,\mathrm{MHz}$. Inset: horn antenna distance and angle with respect to vapor cell; all fields linearly polarized along the *z*-axis. EIT peak height (**b**, **d**) and FWHM (${\varGamma }_{{EIT}}$) (**c**, **e**) as a function of probe (**b**, **c**) or coupling (**d**, **e**) laser intensity/Rabi frequency, fits detailed in the text. **f** Measured EIT signals at three coupling laser Rabi frequencies showing the asymmetric broadening at high ${\varOmega }_{C}$. Error bars given by $k\sigma$ where $k=2.78$ (95% confidence of 5 measurement repeats) and $\sigma$ is measurement standard deviation. Shaded areas of fits denote 95% prediction bounds

A typical system for the absolute measurement of an RF electric field amplitude using Rydberg Cs atoms is depicted in Fig. 6a. We used this system to study the fabricated glass vapor cells and characterize their performance, finding that these wafer-processed cells indeed support the measurement of millimeter wave fields and show minimal deleterious effects from charging or stray fields within the cells.

We excite the atoms to the 58D_5/2_ Rydberg state and step either the probe or the coupling laser power while monitoring the EIT spectrum. In Fig. 6, we plot the resulting EIT peak height (b, d) and full width at half-maximum (FWHM) (c, e) as the probe (b,c) or the coupling (d, e) laser power (reported as intensity or Rabi frequency) increases. Given the Gaussian intensity profile of the probe laser, the peak intensity (${I}_{P}$ or ${I}_{C}$) is given by

1 $${I}_{P,C}=\frac{2{P}_{P,C}}{\pi {w}_{P,C}^{2}}$$

where ${P}_{P,C}$ is the probe ($P$) or the coupling ($C$) power prior to entering the vapor cell and ${w}_{P}=350\,{\rm{\mu }}{\rm{m}}$, ${w}_{C}=360\,{\rm{\mu }}{\rm{m}}$ is the width of each Gaussian beam as it passes through the cell. As seen in Fig. 6b, the EIT peak height increases with the probe intensity and saturates. This is determined by fitting the expression for a resonant two-state system:

2 $${h}_{{EIT}}\left({I}_{P,C}\right)=\frac{{h}_{\max }{I}_{P,C}}{\left({I}_{{sat}}+{I}_{P,C}\right)}$$

where ${h}_{\max }$ corresponds to the maximum height of the EIT peak in arbitrary units, and ${I}_{{sat}}$ is the saturation intensity. The fitted values are ${h}_{\max }=7.23\left(4\right)$ a.u., ${I}_{{sat}}=12.3\left(2\right){\rm{mW}}/{\rm{c}}{{\rm{m}}}^{2}$ for the supported cell and ${h}_{\max }=6.34\left(5\right)$ a.u. and ${I}_{{sat}}=13.9\left(2\right){\rm{mW}}/{\rm{c}}{{\rm{m}}}^{2}$ for the open cell when the coupling laser power is held fixed at ${P}_{C}\approx 6\,\mathrm{mW}$ (${\varOmega }_{C}/2\pi \approx 0.7\,\mathrm{MHz}$). Throughout this paper, we report expanded uncertainties with 95% confidence intervals. The inset within Fig. 6b displays a typical EIT signal at one probe intensity, illustrating the characteristic transmission peak that defines EIT. Similarly, the EIT peak height increases with the coupling laser and saturates at an intensity of $2.8\left(1\right){\rm{W}}/{\rm{c}}{{\rm{m}}}^{2}$ with ${h}_{\max }=3.89\left(3\right)$ a.u. when the probe laser power is held fixed at ${P}_{P}\approx 20\,{\rm{\mu }}{\rm{W}}$ (${\varOmega }_{P}/2\pi \approx 9.3\,\mathrm{MHz}$) as shown in Fig. 6d.

In Fig. 6c, e, the input power of the probe or coupling laser is reported as the Rabi frequency, where

3 $${\varOmega }_{P,C}=\frac{{\mu }_{12,23}}{\hslash }\left|{E}_{P,C}\right|$$

${\mu }_{12}$ is the dipole moment of the $|6{S}_{1/2}\rangle \left(F=4\right)$ $\to |6{P}_{3/2}\rangle \left(F=5\right)$^43^, ${\mu }_{23}$ is the dipole moment of the $|6{P}_{3/2}\rangle \left(F=5\right)\to |58{D}_{5/2}\rangle$, ${E}_{P,C}=\sqrt{2{I}_{P,C}/c{\varepsilon }_{0}}$, $\hslash$ is the reduced Planck constant, $c$ is the speed of light in vacuum^44^, and ${\varepsilon }_{0}$ is the vacuum permittivity. The EIT linewidth (${\varGamma }_{{\rm{EIT}}}$), defined as the FWHM, increases with probe Rabi frequency, showing the expected power broadening trend which can be modeled by

4 $${\varGamma }_{{EIT}}\left({\varOmega }_{P}\right)=\sqrt{A{\left({\varOmega }_{P}/2\pi \right)}^{2}+{\varGamma }_{0}^{2}}+{\varGamma }_{P}$$

where ${\varGamma }_{0}/2\pi =3.72\,\mathrm{MHz}$ accounts for the lifetime of the Rydberg state, collisions, transit time broadening, and residual Doppler broadening (dominant). The extracted zero-power linewidth is ${\varGamma }_{P}/2\pi =3.9\left(2\right)\,\mathrm{MHz}$ for the supported cell and ${\varGamma }_{P}/2\pi =7.4\left(2\right)\,\mathrm{MHz}$ for the open cell. Similar values of the unitless linear fit coefficient $A$ are measured for the two cells and agree with the expected value of 2^45^: $2.21\left(5\right)$ (supported) and $2.24\left(6\right)$ (open). As the coupling laser power is increased, the EIT linewidth again increases, but the cause in this case is not exclusively power broadening^42^.

In Fig. 6f, three spectra are given showing the EIT signal at three coupling laser Rabi frequencies with the location of the 58D_3/2_ and 58D_5/2_ Rydberg states denoted by the vertical lines. At the highest coupling Rabi frequency, the EIT signals not only broaden but also shift slightly to the right. This is due to Stark shifting caused by a DC electric field induced by the strong coupling laser as it interacts with adsorbed Cs on the surface of the windows. Each momentum substate ${m}_{J}$ undergoes different amounts of shift due to differences in polarizabilities. Furthermore, the electric field across the region of Rydberg atoms is not likely homogeneous. The integrated effect is the broadening of the EIT signal. Given enough coupling laser power, we expect to be able to resolve the ${m}_{J}$ substates as in ref. ^46^ even with the expected inhomogeneous field that we integrate over the 2 mm length of the cell. The exact dependence of this induced electric field on coupling laser power density along with accounting for the field inhomogeneity is difficult to know. We, therefore, choose a simple model that accounts for saturation of the induced field (see Section “Coupling laser dependent broadening”) for the EIT linewidth dependence on coupling laser Rabi frequency in Fig. 6e:

5 $${\varGamma }_{{EIT}}\left({\varOmega }_{C}\right)=\sqrt{B{\varOmega }_{C}^{4}+{\varGamma }_{0}^{2}}+{\varGamma }_{C}$$

see Section “Coupling laser dependent broadening” for a more detailed justification for this model. Using this model, the zero-power linewidth is estimated as ${\varGamma }_{C}=8.4\left(2\right)\,\mathrm{MHz}$, which is in part caused by power broadening from the probe laser (${\varOmega }_{P}/2\pi \approx 9.3\,\mathrm{MHz}$), and the power-scaling fit coefficient is found to be $B=4.6\left(1\right)$ (notably larger than $A\approx 2$ in Eq. 4).

Next, we introduced a resonant millimeter wave field to drive transitions between the 58D_5/2_ and 60 P_3/2_ Rydberg states of the Cs atoms to observe Autler-Townes splitting. To do this, a 34 GHz millimeter wave signal is generated and amplified then radiated on to the Cs vapor cell using a horn antenna positioned $\approx 57$ cm from the cell at an angle of $\approx {12}^{\circ }$ from the plane of the thin vapor cell (i.e., $\approx {78}^{\circ }$ from the axis of the lasers, which are approximately normal to the window surfaces of the vapor cell), see Fig. 6a for a diagram of the setup. This geometry was simply practical for our optical table setup and not suggestive of a unique behavior of these cells. The millimeter wave field strength is ramped by adjusting the input power defined at the generator, allowing us to study the effect of varying millimeter wave field amplitudes on the atomic transitions. The dielectric structure of the cells, either open or supported, modifies the RF fields inside the atomic volumes, which must be modeled and taken into account in RF metrology applications. For frequencies used in this manuscript, the cell structures are much smaller than the RF wavelength and no resonant RF field enhancement is expected, while modest and broadband field modulation due to cell reflection and near-field electrostatic-limit enhancement may be present, dependent on polarization.

**Fig. 7:**
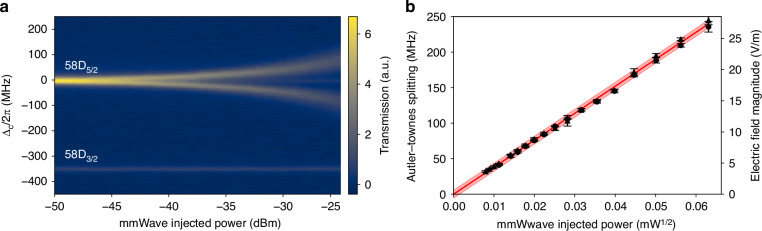
Rydberg atom electrometry. **a** Autler-Townes splitting of the 58D_5/2_ Rydberg state is apparent when a 34 GHz electric field, resonant with 60 P_3/2_, is applied with increasing power fed to the horn antenna. **b** Frequency separation between the split states (${\Delta }_{{AT}}$) increases linearly with millimeter wave field strength ($\propto \sqrt{{Power}}$). These measurements were taken with an open cell

The experimental results illustrating Autler-Townes (AT) splitting are presented in Fig. 7. Figure 7a shows the transmission spectrum as a function of millimeter wave power defined at the generator and the detuning of the coupling laser (${\Delta }_{C}$). As the millimeter wave power increases, a clear splitting of the EIT resonance becomes evident. This splitting corresponds to the Autler-Townes effect between the 58D_5/2_ and 60 P_3/2_ states, confirming the strong coupling induced by the millimeter wave field. The color scale represents the transmission intensity in arbitrary units (a.u.), and both the splitting and the broadening of the spectral lines become more pronounced with higher millimeter wave power. Broadening of the AT lines at high power is caused by inhomogeneities in the millimeter wave field within the cell^47^ and m_*j*_ sublevel splitting of the Rydberg state. These sublevels are degenerate but become visible under a strong applied electric field due to differences in the polarizabilities of each state^48^.

To quantitatively analyze Autler-Townes splitting, we measure splitting ${\Delta }_{\mathrm{AT}}$ as a function of the input millimeter wave power to an amplifier and horn antenna, as shown in Fig. 7b. The data exhibit a linear relationship between ${\Delta }_{\mathrm{AT}}$ and the square root of the millimeter wave power, which aligns with the theoretical expectation that this splitting is equal to the Rabi frequency of the applied millimeter wave field (${\varOmega }_{{mm}}$) and is proportional to the electric field amplitude (${E}_{{mm}}$) of the millimeter wave (${\varOmega }_{{mm}}\propto {E}_{{mm}}\propto \sqrt{{P}_{{mm}}}$, where ${P}_{{mm}}$ is the millimeter wave power). A linear fit to the data yields a calibration factor of $3797\left(6\right){\rm{MHz}}/\sqrt{{\rm{mW}}}$. The right axis of Fig. 7b gives the corresponding electric field amplitude in volts per meter calculated using the relation

6 $${E}_{{mm}}=\frac{\hslash }{{\mu }_{34}}{\Delta }_{\mathrm{AT}}$$

where ${\Delta }_{\mathrm{AT}}$ is the measured Autler-Townes splitting, and ${\mu }_{34}$ is the transition dipole moment between the Rydberg states. For the $|58{D}_{5/2}{\rm{\rangle }}\to |60{P}_{3/2}\rangle$ transition, the dipole moment is calculated to be ${\mu }_{34}=685\cdot e\cdot {a}_{0}$^49^, where $e$ is the charge of an electron and ${a}_{0}$ is the Bohr radius.

## Discussion

The wafer-level fabrication process presented here demonstrates an all-glass approach to creating millimeter- to centimeter-scale vapor cells suitable for room temperature atomic spectroscopy measurements, including Rydberg atom electrometry. The use of Borofloat 33^32^, which has a significantly lower dielectric constant and loss tangent compared to silicon, exemplifies the potential that this direct bonding fabrication approach offers, which is an extending of vapor cell material options for specific application needs.

Our results confirm that these wafer-processed glass vapor cells maintain vacuum integrity, support reproducible alkali loading, and provide reliable Rydberg electrometry at 34 GHz. Specifically, the observed EIT spectra are robust over several months, which confirms that the Cs activation process adequately fills the cells and the hermetic sealing has no apparent leak. The measured SAS and EIT linewidths are on par with those of other microfabricated vapor cells^21,22,25,50,51^. From the dependencies in Fig. 6c, e and recognizing the known Zeeman splitting^9^ and photo-induced DC Stark shifting^46^, our analysis goes a step further than other works by attempting to identify all sources of line broadening. The Autler–Townes splitting measurements show a clean linear dependence of the splitting on the square root of the incident millimeter wave power, consistent with theoretical expectations. This linear relationship offers a direct calibration of the electric field amplitude at the atoms and emphasizes the suitability of these fabricated cells for quantifying millimeter wave fields at frequencies relevant to next-generation communication and radar systems.

There are several ways to improve and extend this platform. Although we rely on borosilicate glass in the present work, the same direct bonding approach can be applied to other low-loss dielectrics (assuming ultraclean surface preparation and flatness), offering greater design flexibility for advanced electromagnetic and optical integration. For example, materials with even lower dielectric constants or lower RF losses^33^ could further increase measurement fidelity in the millimeter wave bands. The ability to make structured cells entirely out of low-loss dielectric materials opens new and exciting possibilities for custom engineered RF-structure interactions, such as sharp and frequency-, angle-, and polarization-specific field enhancement inside the cell, as well as customized far-field scattering modification. Such dielectric cells may also be better suited for high RF power density applications. Additionally, refining the internal cell geometry may allow for tailored mode confinement or specialized optical paths crucial for multiaxis or high-spatial-resolution sensing.

A reaction reservoir, as is commonly found in wafer-processed vapor cells, would also minimize the collection of alkali metal on the windows of the cell. Given that this thin metal film is known to interact with the coupling laser used in two-photon Rydberg EIT systems causing stray electric fields that may broaden and shift the EIT signal^46^, it is beneficial to minimize the adsorption of Cs on the surfaces of the cell where the lasers propagate. Furthermore, an alkali getter has been shown to cause perturbations to an incident RF field^25^. A make-seal technique like that presented by Maurice et al. may even allow complete removal of the reaction chamber^52^. Studies of the spatial variation in both the EIT signal of a set of Rydberg states and the AT splitting to inform millimeter wave field uniformity across the vapor cells discussed here are under way and will be the subject of a future publication.

Although we demonstrate a robust batch-fabrication technique on wafer, future research could focus on the integration of photonic components such as waveguides, gratings, on-chip heaters, or even on-chip lasers. Such platforms promise to reduce device footprints while enabling wide-scale deployment in industrial, defense, and medical applications where high-accuracy field sensing or atomic spectroscopic tools are desired. Overall, our all-glass direct bonding process lays the groundwork for a new generation of millimeter-scale atomic vapor cells, providing precise Rydberg electrometry without the material constraints posed by silicon.

We demonstrate an all-glass wafer-level fabrication process for millimeter-scale vapor cells that enables reliable Rydberg atom electrometry. Our results demonstrate stable vacuum-sealing and alkali-loading processes, validated by the robust and repeatable Rydberg EIT signals measured over many months. We characterize the EIT signal at 58D_5/2_ showing 12.0(2) MHz minimum *measured* linewidth and develop a case for the dominant contributing factors causing this linewidth being residual Doppler broadening and laser-induced broadening. The dependence of the linewidth with coupling laser power suggests that this effect may be largely due to ionization of adsorbed Cs on the windows. Autler–Townes splittings at 34 GHz exhibits the expected linear dependence on the square root of the input microwave power, underscoring the suitability of these cells for precise electric field calibration.

Looking forward, this fabrication approach paves the way for miniaturized, low-loss vapor cells suitable for high-frequency field sensing and offers flexibility in material choice for future systems. We plan to explore alternative cell geometries, integrated photonic components, internal coatings, and activation reservoirs to further enhance long-term alkali control and minimize stray fields. Ultimately, these improvements will broaden the applicability of Rydberg-based sensors for radar, communication, and metrology, paving the way toward fully integrated, mass-manufactured, miniaturized quantum devices that operate at higher frequencies and with higher spatial resolution.

## Materials and methods

### Vapor cell geometry details

The internal volume of each vapor cell is machined using femtosecond laser pulses and KOH etching to create either one 19 mm × 13 mm open area reservoir or seven 19 mm long by 1.3 mm tall horizontal trenches that are connected by a single 1 mm wide vertical trench. The total evacuated volume of each open cell is $494\,{\rm{m}}{{\rm{m}}}^{3}$ and the total evacuated volume of each supported cell with trenches is $354.2\,{\rm{m}}{{\rm{m}}}^{3}$. This trench geometry minimizes bowing of the vapor cell windows to facilitate multiple reflections of the lasers within the cell over long path lengths without alignment errors (see Section “Explanation of cell geometry” for detailed discussion of this vapor cell geometry).

### Atomic spectroscopy measurements

In this system, the ground state, $|6{S}_{1/2}{\rm{\rangle }}\left(F=4\right)$, is coupled to the intermediate state, $|6{P}_{3/2}{\rm{\rangle }}\left(F{\prime} =5\right)$, via a weak probe laser at 852.3 nm. Simultaneously, the transition between the intermediate state and the Rydberg state, $|58{D}_{5/2}{\rm{\rangle }}$, is driven by a strong coupling laser field at 509 nm. The probe laser is generated using a grating-stabilized tunable single-mode laser, while the coupling laser is produced by a frequency-doubled tunable diode laser system capable of delivering up to 300 mW of power. The probe laser is power stabilized with an electronically controlled variable optical attenuator (EVOA), and a pair of acousto-optic modulators (AOMs) are used to power stabilize and modulate the coupling laser beam at a frequency of 37.2 kHz. The lasers are aligned to counterpropagate through the Cs vapor cell at room temperature with the lasers approximately normally incident on the cell windows. The probe laser beam width is $350{\rm{\mu }}{\rm{m}}$ and the coupling laser beam width is $360{\rm{\mu }}{\rm{m}}$ through the 2 mm vapor cell length. The EIT signal is recorded through differential detection, incorporating a balanced photodetector to minimize common noise in the probe laser with a reference beam that also passes through the vapor cell. The detected signal is then fed into a lock-in amplifier, synchronized with the AOM modulation, to extract the EIT signals with improved signal-to-noise ratio. The lock-in amplifier is configured with a time constant of 30 μs, a sensitivity of 200 mV (typical), and 24 dB setting for the low pass filter roll-off. Additionally, the frequency of the probe laser is stabilized using a reference ultra-low expansion Fabry-Perot cavity.

To capture the EIT spectra reported in Figs. 6 and 7, we scan the frequency of the coupling laser over the 58D_3/2_ and 58D_5/2_ Rydberg states, observing increased probe laser transmission when the coupling laser is near resonance with each state. The calculated frequency difference between the 58D_3/2_ and 58D_5/2_ states is 351.21 MHz^49^ and sets the frequency axis of the laser scan. In this experiment, the RF signal is generated by a signal generator (up to 40 GHz), which provides a stable continuous wave microwave output at 34.009 GHz. The output of the signal generator is routed into a traveling wave tube amplifier (TWT), which delivers the high gain and power necessary to drive AT splitting of the Rydberg state. A WR-28 rectangular waveguide gain horn efficiently couples the amplified microwave signal into free space to illuminate the vapor cell. The combined TWT gain and coax cable and connector loss between the signal generator output connector and the coax connector at the input of the horn antenna is $\approx$39.5 dB and the specified antenna gain is 23 dBi.

### Coupling laser dependent broadening

The dependence of the EIT linewidth on the coupling laser power is challenging to model analytically, given an inhomogeneous DC electric field within the cell, which scales with the coupling laser power density on the cell window. As demonstrated by Patrick, et al.^46^, the field within the vapor cell exhibits a saturating trend with increasing coupling laser power on the cell surface. We attribute this behavior to the photoelectric effect, where the incident laser generates a charge density on the cell windows, resulting in a non-uniform field across the optical length of the vapor cell. The number of photons in the laser is $n=P/\left({hf}\right)$, where $P$ is the laser power, $h$ is the Planck constant, and $f$ is the laser frequency. At low photon numbers, the number of charges generated is proportional to the number of photons hitting the glass per unit time, where we introduce an efficiency and recombination rate scaling factor ($\eta$). Given the observed saturation of the field (i.e., the generated charges), the total charge generated is then given by ${n}_{q}=\eta n/\left(1+n/{n}_{{sat}}\right)$. The electric field emitted by the surface charges is estimated as $E=k/{r}^{2}\int \sigma {dA}={n}_{q}q/4\pi {\varepsilon }_{0}{r}^{2}$. Substituting the expressions for ${n}_{q}$ and $n$, we obtain

7 $$E=\frac{{qP}\eta }{4\pi {\varepsilon }_{0}{r}^{2}{hf}\left(1+P/{P}_{{sat}}\right)}$$

The Stark effect induces a shift, ${\Delta }_{s}=\alpha {\left|E\right|}^{2}/4$, which leads to broadening due to the variation of the displacement vector magnitude ($r$) within the vapor cell for the set of Rydberg atoms probed. To first order, we assume symmetry between the entrance and exit windows. By substituting the Rabi rate for the optical field, we derive the average shift across the vapor cell as

8 $${\Delta }_{{avg}}=-\frac{\alpha {\eta }^{2}{q}^{2}{c}^{2}{w}_{C}^{4}{h}^{2}}{3072{f}^{2}{\mu }^{4}{r}^{3}}{|}_{{r}_{0}}^{r{\prime} }\cdot {\left(\frac{{\varOmega }_{C}^{2}}{1+{\varOmega }_{C}^{2}/{\varOmega }_{{sat}}^{2}}\right)}^{2}={C}_{1}\cdot {\left(\frac{{\varOmega }_{C}^{2}}{1+{\varOmega }_{C}^{2}/{\varOmega }_{{sat}}^{2}}\right)}^{2}$$

where ${C}_{1}$ encompasses the constants, quantum efficiency, and non-uniformity. The total effect of the coupling laser on the line shape is then described by

9 $$\begin{array}{c}{\varGamma }_{{EIT}}\left({\varOmega }_{C}\right)=\sqrt{{({C}_{1}\frac{{\varOmega }_{C}^{4}}{{\left(1+{\varOmega }_{C}^{2}/{\varOmega }_{{sat}}^{2}\right)}^{2}})}^{2}+{C}_{2}{\left({\varOmega }_{C}/2\pi \right)}^{2}+{\varGamma }_{0}^{2}}\end{array}$$

where ${C}_{2}\ll {C}_{1}^{2}$ is a fit coefficient associated with the coupling laser power broadening term that can be ignored for ${\varOmega }_{C}/2\pi < 15\,\mathrm{MHz}$ at the Rydberg state probed in these measurements. This model predicts an ${\varOmega }_{C}^{8}$ scaling inside the square root in the linear regime and an ${\varOmega }_{C}^{4}$ scaling near saturation. The coupling laser power sweep study reported in Fig. 6d–f is near the saturation power associated with the saturation of the photo-induced electric field such that measured EIT linewidths fit a model that scales as ${\varOmega }_{C}^{4}$ inside the square root.

### Explanation of cell geometry

The specific vapor cell geometry is chosen to enable the construction of a sensor array. In that system, the coupling laser is internally reflected within the vapor cell along the long (X) axis to overlap a one-dimensional array of probe lasers. This internal reflection technique maximizes the power efficiency of the coupling laser; however, alignment of the two lasers is exceptionally susceptible to any non-planarity of the vapor cell windows supporting the mirror coatings.

**Fig. 8:**
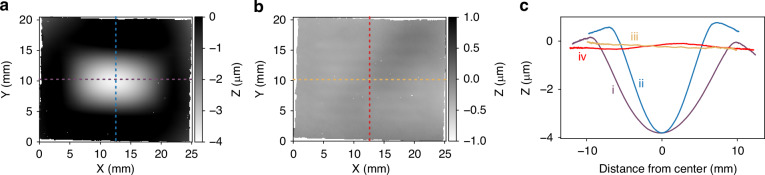
Vapor cell geometry characterization. **a** Height profile of one vapor cell window without glass supports (the open cell). **b** Height profile of one supported cell. **c** Slices along the *X* axis (i and iii) and along the *Y* axis (ii and iv) through the center of each celli and ii correspond to (**a**), the open cell without supports, and iii and iv correspond to (**b**), the cell with supports, as indicated by the colored dashed lines

The pressure difference between the evacuated volume of the vapor cell and atmosphere causes the windows of the cell to bow inwards, Fig. 8a. The dimensions of the evacuated region in this open, non-supported cell is 13 mm tall (Y) by 19 mm wide (X) by 2 mm thick (Z). The maximum deflection of the outer surface of one window is measured to be about $3.8\,{\rm{\mu }}{\rm{m}}$. Given this deflection, we estimate that the position error of the internally reflected coupling beam in our desired architecture would be greater than $130\,{\rm{\mu }}{\rm{m}}$ over the 19 mm cell length given the 9.45° designed incidence angle. This position error is nearly a beam width meaning that array elements at the end of each row would have weak to no signal due to severe misalignment of the two counter-propagating lasers. On the other hand, the window deflection of the vapor cell design with glass support structures (Fig. 8b) is $\ll 1{\rm{\mu }}{\rm{m}}$, such that any alignment error caused by curvature in the windows is negligible. The maximum deflection within an open trench within the supported cell is at the edge of the tool resolution and is estimated to be less than $100\,\mathrm{nm}$ from the height profile data shown in Fig. 8b.

## Data Availability

All of the data presented in this paper and used to support the conclusions of this article is published under the identifier 10.18434/mds2-3762.
